# Asthma and allergic rhinitis: Linked in treatment and outcomes

**DOI:** 10.4103/1817-1737.62467

**Published:** 2010

**Authors:** David Price

**Affiliations:** *General Practice Airways Group, Department of General Practice and Primary Care, University of Aberdeen, Foresterhill Health Centre, Westburn Road, Aberdeen, UK*

Asthma and allergic rhinitis (AR) frequently coexist, with epidemiological data suggesting that most asthma patients also suffer from AR.[1] We also find similar trends in asthma and AR prevalence around the world using objective epidemiological instruments.[2] Both diseases share similar triggers and similar pathophysiology characterized by similar inflammatory cell infiltrates.[3] It has also been found that the degree of inflammation in asthma correlates highly with the level of inflammation in the nose,[4] and that allergen challenge of the nose leads to hyperresponsiveness in the lungs[5] and allergen challenge in the lung with inflammation in the nose.[6] This and other evidence suggests a central mechanism behind the link with eosinophil precursors emanating from the bone marrow in response to triggers migrating not only to the site of stimulation, such as the nasal mucosa, but also to other sites within the one airway, including the lower respiratory tract.

Three areas of research suggest there is a real importance clinically in the ‘one-airway’ concept. The first of these relates to the possibility of interference in the development of asthma in patients with rhinitis; the second is that the severity of rhinitis appears to be a risk factor for poor asthma control and thirdly that common anti-inflammatory therapy may improve outcomes for both.

Examining the first of these, one study from the United States showed that those students with rhinitis in college were up to three times more likely to develop asthma by the age of 40 than those without rhinitis.[7] Intriguingly, Johnstone and Möller observed that young patients with rhinitis receiving subcutaneous immunotherapy for their rhinitis were less likely to develop subsequent asthma suggesting that intervention in patients with rhinitis might reduce the likelihood of developing asthma.[8]

Regarding the second of these, it has been found that clinically diagnosed AR is associated with significantly worse asthma control in adults and children. A recent analysis of patients in a General Practice database in the UK found that children with physician-diagnosed AR were more than twice as likely to be hospitalized for asthma as those without and 50% more likely in adults.[9,10] Studies on children hospitalized for asthma in Norway showed that those children with clinically recognized rhinitis had a greater risk of rehospitalization for asthma.[11] In addition, a recent evaluation of asthma control in general practice suggested that those with asthma and moderate-to-severe rhinitis were four times more likely to have poor asthma control than those with no rhinitis symptoms.[12]

Considering the third one, treatment guidelines have recognized that asthma and AR are linked conditions of ‘One Airway’ and recommend that patients with asthma be evaluated for AR and vice versa. These guidelines support a combined approach to treating both conditions.[3] Such strategies with our current range of therapeutic options include:

**Upper airway treatment options**

Nasal steroids

Antihistamines

**Lower airway treatment options**

Inhaled steroids

**Upper and lower airway treatment options**

Leukotriene receptor antagonists

Anti-IgE

Immunotherapy

What evidence beyond a common-sense approach do we have to support this approach? Many of the studies that have supposedly set out to test this hypothesis have unfortunately been flawed, in that they have often only looked at using treatment for one part of the airway. For example, we have many studies of nasal steroids in patients with rhinitis and asthma but not used in conjunction with inhaled steroids to treat the asthma – these studies in seasonally triggered rhinitis and asthma have been examined in a recent meta-analysis and showed minimal impact on asthma outcomes – which is unsurprising and neither refute nor support the one-airway approach.[13] Because of this lack of appropriate, randomized, controlled trials we have until recently had to rely on data from observational studies relating to the use of antihistamines and nasal steroids. One of these studies used the MarketScan database in Washington, USA, that examined patients with asthma and rhinitis treated for concomitant rhinitis compared to those only treated for their asthma and found asthma-related events were reduced by 49% in those treated for both conditions.[14] Whilst certainly supporting the one-airway approach, randomized trials are needed to confirm these findings.

The advent of treatments that both treat the upper and lower airways provides an opportunity to examine this in a different way. These include drugs that block IgE, cysteinyl leukotrienes and the use of immunotherapy as all three approaches have evidence to support their usage in patients with asthma and AR.[15–19] The ideal study design would compare the benefits of a sole lower airway approach with a combined approach in patients with asthma alone and asthma with rhinitis. The only analysis that has set out to do this to date is that of the COMPACT study. The COMPACT study was a randomized, double-blind trial of 889 adults with asthma, whose asthma symptoms persisted despite being prescribed inhaled steroids with groups randomized to an increase in inhaled steroids or addition of a leukotriene receptor antagonist.[20] Further analysis examined the response in patients with and without a physician and patient report of rhinitis. This showed that those patients with asthma and no rhinitis had similar improved outcomes irrespective of the type of treatment increase; however, those with rhinitis showed a greater benefit with a one-airway approach of using upper and lower airways treatment with inhaled steroids and a leukotriene antagonist. This benefit was predominantly produced because of a reduced efficacy from increasing inhaled steroids in this subgroup.[21]

In conclusion, AR and asthma are inflammatory disorders that have been linked epidemiologically, pathophysiologically and clinically. Allergic rhinitis increases morbidity, therapeutic needs and use of healthcare resources in patients with asthma. It is, therefore, important to recognize co-morbidity in patients with asthma and consider management strategies from this perspective; failure to do so appears to result in adequate asthma control.
